# Investigating the Role of Interpretation Bias in Mindfulness-Based Treatment of Adults With Generalized Anxiety Disorder

**DOI:** 10.3389/fpsyg.2020.00082

**Published:** 2020-02-11

**Authors:** Elizabeth A. Hoge, Hannah E. Reese, Isabelle A. Oliva, Caroline D. Gabriel, Brittany M. Guidos, Eric Bui, Naomi M. Simon, Mary Ann Dutton

**Affiliations:** ^1^Department of Psychiatry, Georgetown University Medical Center, Washington, DC, United States; ^2^Department of Psychology, Bowdoin College, Brunswick, ME, United States; ^3^Department of Psychiatry, Center for Anxiety and Traumatic Stress Disorders, Massachusetts General Hospital, Boston, MA, United States; ^4^Department of Psychiatry, New York University Langone Health, New York, NY, United States

**Keywords:** anxiety, Generalized Anxiety Disorder, cognitive bias, homophone, interpretation bias, mindfulness

## Abstract

Although mindfulness-based interventions (MBIs) have garnered empirical support for a wide range of psychological conditions, the psychological processes that mediate the relationship between MBIs and subsequent symptomatic improvement are less well-understood. In the present study we sought to examine, for the first time, the relationship between mindfulness, negative interpretation bias as measured by the homophone task, and anxiety among adults with Generalized Anxiety Disorder (GAD). Forty-two individuals with GAD completed measures of mindfulness, interpretation bias, and anxiety before and after treatment with Mindfulness-based Stress Reduction (MBSR). Contrary to prior research, we did not find evidence of an indirect relationship between baseline levels of mindfulness and anxiety via negative interpretation bias. MBSR did result in significant reductions in negative interpretation bias from baseline to post-treatment; however, we did not find evidence of an indirect relationship between changes in mindfulness and changes in anxiety via changes in interpretation bias. Taken together, these results provide minimal support for the hypothesized relationship between mindfulness, negative interpretation bias, and anxiety among adults with GAD. Limitations and specific suggestions for further inquiry are discussed.

## Highlights

- We examined the role of interpretation bias in the mindfulness-based treatment of adults with GAD.
- Participants experienced significant reductions in mindfulness, negative interpretation bias, and anxiety.
- We did not find evidence for an indirect relationship between mindfulness and anxiety via interpretation bias.

## Introduction

Mindfulness-Based Interventions (MBIs) aim to increase awareness of present-moment experiences, including thoughts, emotions, and bodily sensations, and the cultivation of a gentle and accepting attitude toward oneself (Bishop et al., 2004). A growing empirical literature supports the efficacy of MBIs to treat a wide range of psychological conditions, such as anxiety and depression (Hofmann and Gómez, 2017). However, the psychological processes that mediate the relationship between MBIs and subsequent symptomatic improvement are less well-understood. A range of different processes including changes in attention, body awareness, emotion regulation, and perspective on the self have been proposed and examined (Holzel et al., 2011). In the present paper, we seek to examine whether changes in another cognitive process, *interpretation bias*, might be one pathway through which Mindfulness-based Stress Reduction (MBSR) produces symptomatic improvement among a sample of adults with Generalized Anxiety Disorder (GAD).

### Evidence for Mindfulness-Based Treatments for GAD

Various researchers have examined the use of MBIs to treat GAD. For example, Evans et al. (2008) recruited 11 subjects (six female, five male) aged 18–80 years old who met DSM-IV criteria for GAD and found that an 8-week MBSR course was effective in reducing anxiety in patients with GAD (Evans et al., 2008). Similarly, Asmaee Majid et al. (2012) recruited 37 male patients aged 25–39 who met criteria for GAD and found that the same 8-week MBSR course improved symptoms of GAD patients (Asmaee Majid et al., 2012). In a previously published trial from which the current study data derive, Hoge et al. (2013) compared an 8-week MBSR course with an attention control condition in 93 DSM-IV-diagnosed GAD patients, and found a significant decrease in several, but not all, anxiety symptoms (Hoge et al., 2013). Wong et al. (2016) recruited 182 participants with GAD who were then assigned to one of three 5-month-long treatment arms, Mindfulness Based Cognitive Therapy (MBCT), Cognitive Behavioral Therapy (CBT) psychoeducation, or “usual care,” and found that MBCT was superior to the other treatment groups in reducing symptoms in GAD.

### Potential Mechanisms of Observed Effects in GAD

The literature examining the psychological processes through which MBI may improve anxiety and worry among adults with GAD is small and nascent. Hoge et al. (2015) found evidence to suggest that increases in one’s ability to decenter, or in other words to observe one’s own thoughts, emotions, and sensations as transient and ever-changing psychological events rather than fixed reflections of reality and the self, mediated the effect of MBSR on anxiety and worry among a sample of adults with GAD. Similarly, Hayes-Skelton et al. (2015) found evidence to suggest that increases in decentering preceded and predicted symptomatic change among adults with GAD who received an acceptance-based behavior therapy. Eustis et al. (2016) found that reductions in experiential avoidance, which is the tendency to control or escape negative internal experiences (e.g., thoughts, emotions, sensations), predicted improvements in worry and quality of life among adults with GAD who received an acceptance-based behavior therapy (ABBT). In the present study, we seek to add to this important literature by examining another candidate process: interpretation bias.

### Interpretation Bias and GAD

Cognitive-behavioral models of anxiety disorders (Hirsch and Mathews, 2012) propose that the way in which an individual interprets ambiguous information in the world around them has direct consequences for their emotional state, physiological arousal, and subsequent behavior. Specifically, individuals with GAD often possess a negative interpretation bias (Hirsch et al., 2016). That is, when compared to non-anxious controls, people with GAD are more likely to interpret ambiguous stimuli as threatening (Bui et al., 2017). Indeed, recent work in which interpretation biases have been experimentally manipulated via cognitive bias modification has demonstrated that interpretation bias may play a causal role in the development and maintenance of anxiety (Hallion and Ruscio, 2011; Menne-Lothmann et al., 2014).

### How Might Mindfulness Influence Interpretation Bias?

Although much of the teaching in MBSR is centered around training one’s attention to present moment experiences, there is also a very strong emphasis on the way or attitude in which one pays attention. Indeed, Kabat-Zinn identified seven attitudinal foundations of mindfulness (non-judging, patience, “beginner’s mind,” trust, non-striving, acceptance, and letting go) which are embedded within the meditative practices and embodied by the teacher. Several of these attitudinal foundations of mindfulness could contribute to the development of a less biased view of the world, such as, non-judging (observing without judging) and “beginner’s mind,” which is conceptualized as a way of seeing the world that is new and uninfluenced by past experiences (Kabat-Zinn, 2016). These concepts are consistent with the idea that over time people develop a biased way of interpreting stimuli in the world, which can be problematic, and a goal of mindfulness training is to reduce or eliminate these biases. The MBSR curriculum includes specific exercises that introduce the idea of ambiguous stimuli that have varied interpretations, such as an ambiguous drawing that can be seen as an old or young woman. These practices aim to help participants identify their implicit assumptions, recognize alternative viewpoints or interpretations, and practice seeing their experiences as they *are* rather than interpreting them through their cognitive filters.

The relationship between mindfulness and interpretation bias has only recently been examined. Mayer et al. (2019) conducted a cross-sectional study examining the relationship between mindfulness, interpretation bias, and symptoms of anxiety in an undergraduate student sample. They found a significant negative relationship between dispositional mindfulness and interpretation bias such that individuals who reported higher levels of mindfulness were less likely to report negative interpretations of ambiguous scenarios. They also found a significant negative relationship between dispositional mindfulness and symptoms of anxiety such that individuals who reported higher levels of mindfulness were less likely to report symptoms of anxiety. Moreover, the authors found evidence for an indirect relationship between dispositional mindfulness and anxiety via interpretation bias. That is, the relationship between dispositional mindfulness and symptoms of anxiety was partially accounted for by negative interpretation bias. These data were cross-sectional, however, so while they are consistent with a causal link between mindfulness, interpretation bias, and anxiety, they should not be over-interpreted. Additionally, the participants were non-clinical undergraduates.

### The Present Study

In the present study, we sought to replicate and extend these findings in a sample of adults with GAD who received treatment with MBSR. MBSR is a standardized, protocolized course that was developed to teach participants how to cultivate mindfulness through a range of meditative practices. This course is now available in most major cities in the United States and has now been used in hundreds of research trials to address a variety of psychological and medical symptoms.

Our first aim was to replicate Mayer et al. (2019)’s cross-sectional findings by examining the indirect relationship between mindfulness and anxiety via interpretation bias at baseline. We hypothesized that we would find evidence of this indirect relationship. Our second aim was to investigate the relationship between mindfulness, interpretation bias, and anxiety over treatment. More specifically, we sought to test for an indirect relationship between changes in mindfulness and changes in anxiety via changes in interpretation bias among adults with GAD receiving MBSR. We hypothesized that there would be an indirect relationship between changes in mindfulness and changes in anxiety via changes in interpretation bias.

## Materials and Methods

### Participants

Participants were 42 individuals with a primary diagnosis of GAD who participated in a clinical trial comparing MBSR (*n* = 42) to an active control condition, Stress Management Education (SME; *n* = 28), for the treatment of GAD in Boston, Massachusetts (for details, please see Hoge et al., 2013). To be eligible for participation in the treatment trial, participants had to meet the following inclusion and exclusion criteria: (1) Diagnostic and Statistical Manual-IV (DSM-IV, American Psychiatric Association [APA], 2000) criteria for current primary GAD, as determined on the Structured Clinical Interview for the DSM-IV (SCID; First et al., 1996), (2) score of at least 20 on the Hamilton Anxiety Rating Scale, indicating clinically significant anxiety and appropriateness for treatment (HAM-A; Shear et al., 2001), (3) no lifetime history of schizophrenia or any other psychosis, mental retardation, organic mental disorders, bipolar disorder, post-traumatic stress disorder or obsessive compulsive disorder, (4) no alcohol or substance abuse or dependence within the past 6 months, (5) no significant suicidal ideation or behaviors within past 6 months, (6) if on medication, on a stable dose for at least 4 weeks, and willing to remain on that dose throughout the study, (7) no serious medical illness or instability, (8) no concurrent psychotherapy directed toward GAD, (9) no more than four classes of meditation training and practice (including yoga and tai-chi) in the past 2 years; (10) not currently pregnant or lactating, and (11) no significant personality disorder likely to interfere with study participation as determined by clinical examination and interview performed by study clinician (MD or Ph.D.). To be eligible for inclusion in this secondary analysis study, participants had to have completed all baseline assessments (as detailed below).

### Intervention

The MBSR class consisted of eight weekly group classes with a single weekend “retreat” day, and daily home practice guided by audio recordings. In-class practices (breath-awareness, a body-scan, and gentle Hatha yoga) were used to cultivate awareness of internal present-moment experiences with an attitude of acceptance and non-judgment. For example, the movement practices contain gentle stretching and slow movements, focusing on present experience and being kind to the body. Participants were also instructed in “informal” home mindfulness practice (e.g., present-focused awareness during eating, bathing, or cleaning).

### Questionnaires and Tasks

The following questionnaires and tasks were administered at baseline (week 0) and endpoint (week 8).

#### Mindfulness

*The Five-Facet Mindfulness Questionnaire* (FFMQ) is a widely used 39-item self-report measure of mindfulness that was empirically derived from a factor analysis of various mindfulness questionnaires. The five facets of mindfulness that emerged from the factor analysis are: describing, observing, acting with awareness, non-judgment, and non-reactivity (Baer et al., 2006). The FFMQ has previously demonstrated good construct validity (Baer et al., 2008). Items are rated on a 5-point Likert-type scale from 1 (never or very rarely true) to 5 (very often or always true). Item scores are summed to generate a total score that can range from 39 to 195 with higher scores indicating greater mindfulness. In the present study, Cronbach’s alpha for the FFMQ at baseline was 0.870.

#### Interpretation Bias

The homophone task was developed by Mathews et al. (1989) to measure biases in interpretation among individuals with GAD. Participants listened to a series of words from a recorded tape and were asked to write down each word that they heard. The words presented included 14 threatening words, 28 neutral words, and critically, 14 homophones. Each of the homophones had two possible spellings: a threatening spelling and a non-threatening spelling (e.g., weak/week; die/dye). The percentage of the homophones spelled in the threatening way was the primary measure of interpretation bias. If an individual listed both spellings, the first spelling provided was included as the response. If a spelling did not represent either of the homophones, then that word was not included in the denominator. The audio recordings were made by a person with an American accent, and all the participants were American.

#### Anxiety

The Beck Anxiety Inventory (BAI) is a widely used 21-item self-report measure of anxiety (Beck et al., 1988). It has previously demonstrated good construct validity and high internal consistency (Fydrich et al., 1992). In the present study, Cronbach’s alpha for the BAI at baseline was 0.795. Each question is multiple-choice and can be answered from 0 (not at all) to 3 (severe) for a total score ranging from 0 to 63. Total scores can then be classified according to severity from minimal to mild, moderate, and severe.

## Results

### Data Reduction

Participants without data for a particular measure were not included in analyses pertaining to that measure. Missing item-level data was imputed to the measure mean. Analyses were conducted using SPSS version 25.

### Baseline Characteristics

Forty-two people with GAD participated. Seventeen (40%) were female, seven (19%) were non-white (Asian, African-American, and other) and the average age was 41.9 (SD = 14.5) years. Summary data for all measures at each time point can be found in Table 1. Additionally, BAI severity scores at baseline ranged from minimal (21.4%) to mild (26.2%), moderate (30.6%), and severe (11.9%).

**TABLE 1:** Summary data for all outcome measures at baseline and post-treatment.

	FFMQ		Homophone task		BAI	
	Mean	*SD*	Mean	*SD*	Mean	*SD*
Baseline	117.79	17.1	0.78	0.13	14.82	8.27
Endpoint	129.18	22.01	0.71	0.15	9.56	7.5

*FFMQ, Five Facet Mindfulness Questionnaire; BAI, Beck Anxiety Inventory. Data for the homophone task represent the percentage of the emotionally ambiguous homophones that were spelled in a threatening manner.*

### Analysis

Aim 1: Investigating the indirect relationship between level of mindfulness and level of anxiety via level of negative interpretation bias at baseline.

To test for the hypothesized indirect relationship, we conducted a simple mediation analysis using ordinary least squares path analysis using the PROCESS macro in SPSS (Hayes et al., 2019). This analysis produces a 95% confidence interval for the indirect effect using 10,000 bootstrapped samples. Evidence of an indirect effect is supported by a confidence interval that does not contain zero. This approach, as argued by Hayes et al. (2019) and others, is superior to the traditional causal steps approach (Baron and Kenny, 1986), preferable in small samples, not dependent on a normal distribution of the underlying data, and provides a more powerful and targeted test of the indirect effect.

**FIGURE 1:**
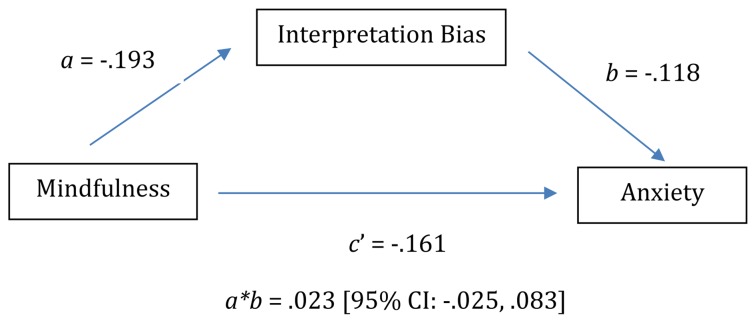
Model depicting baseline indirect path analysis of relationship between mindfulness, negative interpretation bias, and anxiety.

Five-Facet Mindfulness Questionnaire total score was included as the predictor, BAI total score was the outcome, and the percentage of homophones interpreted in a negative manner was the mediator. In contrast to Mayer, we did not find evidence of a significant indirect effect of mindfulness on anxiety via negative interpretation bias. The bootstrap confidence interval for the indirect effect (*ab* = 0.023) based on 10,000 bootstrap samples contained zero (-0.025 to 0.083). Indeed, the only significant relationship we observed was the direct effect of mindfulness on anxiety when holding interpretation bias constant (path *c’* in Figure 1). Two individuals who differed by one unit of mindfulness, but who demonstrated the same level of interpretation bias, would differ by 0.161 points in anxiety such that higher mindfulness is related to lower anxiety (see Figure 1 and Table 2 for complete details of the model).

**TABLE 2:** Model estimates for indirect path analysis of relationship between mindfulness, negative interpretation bias, and anxiety at baseline.

		Outcome						
		M (Interpretation bias)				Y (Anxiety)		
Predictor		Coeff.	SE	*p*		Coeff.	SE	*p*
X (Mindfulness)	*a*	–0.193	0.123	0.124	*c’*	–0.161	0.078	0.046
M (Interpretation bias)		–	–	–	*b*	–0.118	0.103	0.261
Constant	*i*_M_	100.391	14.606	<0.001	*i*_Y_	42.909	13.700	0.004
		*R*^2^ = 0.066				*R*^2^ = 0.121		
		*F*(1,35) = 2.480, *p* = 0.124				*F*(2,34) = 2.341, *p* = 0.112		

Aim 2: Investigating the relationship between mindfulness, interpretation bias and anxiety over treatment.

We first conducted three separate two-tailed paired *t*-tests to examine whether mindfulness, interpretation bias and anxiety changed over treatment. As expected, participants did indeed exhibit a significant increase in mindfulness, *t*(37) = −3.629, *p* = 0.001, *d* = −0.54, decrease in negative interpretation bias, *t*(41) = 2.778, *p* = 0.008, *d* = 0.43 and decrease in anxiety, *t*(35) = 4.055, *p* < 0.001, *d* = 0.68 from baseline to endpoint.

**FIGURE 2:**
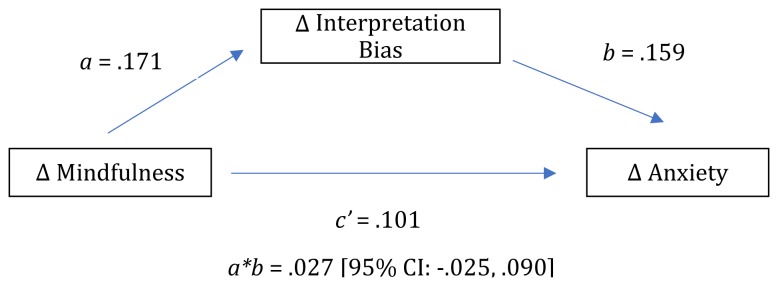
Model depicting indirect path analysis of relationship between change in mindfulness, negative interpretation bias, and anxiety.

To test for the hypothesized indirect relationship between changes in mindfulness and changes in anxiety via changes in level of negative interpretation bias from baseline to post-treatment, we again conducted a simple mediation analysis using ordinary least squares path analysis. In this case, we included change in total FFMQ score as the predictor, change in total BAI score as the outcome, and change in the percentage of homophones interpreted in a negative manner as the mediator. Again, contrary to our hypotheses, we did not find evidence of a significant indirect effect of changes in mindfulness on changes in anxiety via changes in interpretation bias. A bootstrap confidence interval for the indirect effect (*ab* = 0.029) based on 10,000 bootstrap samples included zero (−0.025 to 0.090; see Figure 2 and Table 3 for complete details of the model). Indeed, the only significant relationship we observed was between changes in interpretation bias and changes in anxiety (path *b* in Figure 2) such that for every one point decrease in negative interpretation bias, participants exhibited a 0.159 point decrease in anxiety.

**TABLE 3:** Model estimates for indirect path analysis of relationship between change in mindfulness, change in interpretation bias, and change in anxiety.

		Outcome						
		M (Interpretation bias change)				Y (Anxiety change)		
Predictor		Coeff.	SE	*p*		Coeff.	SE	*p*
X (Mindfulness change)	*a*	0.171	0.141	0.232	*c’*	0.101	0.065	0.131
M (Interpretation bias change)		–	–	–	*b*	0.159	0.078	0.0499
Constant	*i*_M_	3.903	3.076	0.213	*i*_Y_	3.181	1.431	0.033
		*R*^2^ = 0.042				*R*^2^ = 0.199		
		*F*(1,34) = 1.482, *p* = 0.232				*F*(2,33) = 4.090, *p* = 0.026		

## Discussion

To our knowledge, this is the first study to examine the relationship between mindfulness, interpretation bias, and symptomatic change with treatment among a sample of well-characterized, treatment-seeking clinically diagnosed adults with GAD. Taken together, our results provide minimal support for a relationship between mindfulness, interpretation bias, and anxiety. We did not replicate Mayer et al. (2019)’s cross-sectional finding of an indirect relationship between mindfulness and anxiety via interpretation bias. That is, negative interpretation bias did not account for the negative relationship between mindfulness and anxiety. MBSR did result in significant reductions in negative interpretation bias. However, we did not find evidence of an indirect effect between changes in mindfulness and changes in anxiety via changes in interpretation bias. That is, our data are not consistent with the hypothesis that changing mindfulness produces changes in interpretation bias that then produce changes in anxiety.

There are several possible reasons why we did not replicate Mayer’s prior results at baseline. One possibility is that there is no relationship between mindfulness, interpretation bias, and anxiety. A second possibility is that there is a relationship between mindfulness, interpretation bias, and anxiety among non-clinical undergraduates, but this finding does not generalize to individuals with clinically significant levels of anxiety and worry. A third possibility is that there is a relationship between mindfulness, interpretation bias, and anxiety in individuals with clinically significant levels of anxiety and worry, but we were unable to detect it. It could be that our sample size was inadequate to detect a small effect. We chose analyses that are particularly well-suited to small samples, but our sample of 42 subjects clearly affords us less power than Mayer’s sample of 133 subjects. It is also possible that our measure of interpretation bias, the homophone task, was less sensitive to individual differences. We chose the homophone task because of its prior use in the literature (Mathews et al., 1989), low demand characteristics, ease of interpretation, and ease of administration. The task was one of many administered as part of the parent RCT so it was imperative that we minimized participant burden. However, the homophone task, in which each item is scored in a binary manner (threatening spelling or not) may have been less sensitive to individual differences than the task used by Mayer et al. (2019). Mayer et al. (2019) presented subjects with 15 ambiguous scenarios and asked them to rate the credibility of a negative and neutral interpretation of the scenario on a scale from 0 to 100. They then computed a bias index by subtracting the credibility of the neutral interpretation from the credibility of the negative interpretation. This measure clearly allows for a wider range of scores and likely produced greater variability within the sample. This greater variability, coupled with a likely wider range of mindfulness and anxiety scores obtained in a broad unselected population, may have allowed them to more readily detect correlations among their measures. Future, larger studies, examining individuals across the spectrum from non-clinical to clinical and incorporating multiple measures of interpretation bias could examine these possibilities.

Mindfulness-based Stress Reduction was associated with a significant reduction in interpretation bias. To our knowledge, this is the first demonstration of this effect. And, in our view, it is particularly noteworthy because interpretation, although central to many cognitive-behavioral interventions for anxiety, is not a focus of MBIs. However, a careful study of the MBSR curriculum reveals that although the program never explicitly references interpretation bias, Kabat-Zinn’s writings on MBSR and its curriculum address it in several ways. For example, throughout the program, participants are encouraged to recognize that when they are operating on “auto-pilot” or acting without conscious awareness that they often only see one perspective and are unaware of alternative points of view. Kabat-Zinn writes, “*Sometimes our thoughts act like dream glasses*…*without knowing it, we are coloring everything, putting our spin on it all*…*But if we take off the glasses, maybe, just maybe, we might see a little more accurately what is actually there*” (Kabat-Zinn, 2005). This instruction, in concert with home practice emphasizing beginners mind, and the suspension of expectations, may encourage cognitive flexibility and openness to alternatives. Thus, it is possible that one of the ways in which MBIs produce symptomatic improvements is via changing interpretation bias. If true, this would represent a point of commonality between the mechanisms of action of MBIs and traditional cognitive behavioral therapies. However, it is also possible that change in interpretation bias is simply a consequence of reductions in anxiety rather than a cause. This interpretation would seem to be supported by our failure to observe an indirect effect between changes in mindfulness and anxiety via interpretation bias. However, future, studies which involve repeated observations of interpretation bias and anxiety over the course of treatment are necessary to establish whether any changes in interpretation bias temporally precede and predict any changes in anxiety.

It is also very likely that there are multiple psychological mechanisms of MBIs for GAD. Future studies designed to simultaneously examine multiple mechanisms of MBIs for anxiety will enable us to more fully understand the independent and additive contributions of these mechanisms.

## Conclusion

A greater understanding of the psychological processes through which MBIs produce symptomatic relief is essential to providing targeted, efficient, and effective care for those in need. In the present study, we examined the role of interpretation bias in the mindfulness-based treatment of adults with GAD. Although interpretation bias has been widely studied as a causal mechanism in the development and maintenance of GAD and is a target of many other treatments for GAD, only one prior study has examined its relationship with mindfulness. The present study provides the first examination of the relationship between mindfulness, interpretation bias, and anxiety in a clinical sample of adults with GAD. Overall, our data provide minimal support for the proposed relationship. Although MBSR did result in significant reductions in negative interpretation bias, we did not find evidence for a cross-sectional indirect relationship between mindfulness and anxiety via interpretation bias or evidence of an indirect effect between changes in mindfulness and changes in anxiety via changes in interpretation bias. Future research involving larger samples spanning the spectrum from non-clinical to clinical with multiple, repeated measures may help to reconcile the conflicting findings in the literature.

## Data Availability Statement

The datasets generated for this study will not be made publicly available as permission was not granted in the consent. Permission to access the data can be made by contacting the corresponding author EH (eah103@georgetown.edu).

## Ethics Statement

The studies involving human participants were reviewed and approved by the Partners Human Research Committee/IRB at MGH. The patients/participants provided their written informed consent to participate in this study.

## Author Contributions

EH and HR conceived and carried out the experiment and wrote the manuscript. IO and CG assisted in the preparation of the manuscript and edited the parts of the revisions. BG, MD, EB, and NS helped to shape the analysis and discussion, provided critical feedback, and provided important contributions to the final manuscript.

## Conflict of Interest

The authors declare that the research was conducted in the absence of any commercial or financial relationships that could be construed as a potential conflict of interest.
